# Supplementary material for: Splice-Junction-Based Mapping of Alternative Isoforms in the Human Proteome
Source: Cell Rep. Author manuscript; Available in PMC 2020 Jan 15. (PMC6961840; doi:10.1016/j.celrep.2019.11.026)
Supplement: 2 [file NIHMS1546469-supplement-2.zip › DF1/PXD006675/VentricularSeptum/VentricularSeptum_34_MINK1_AATAAGAGAAEAAGEGAAAAAGGHAGSAAGGGAAAG.pdf]

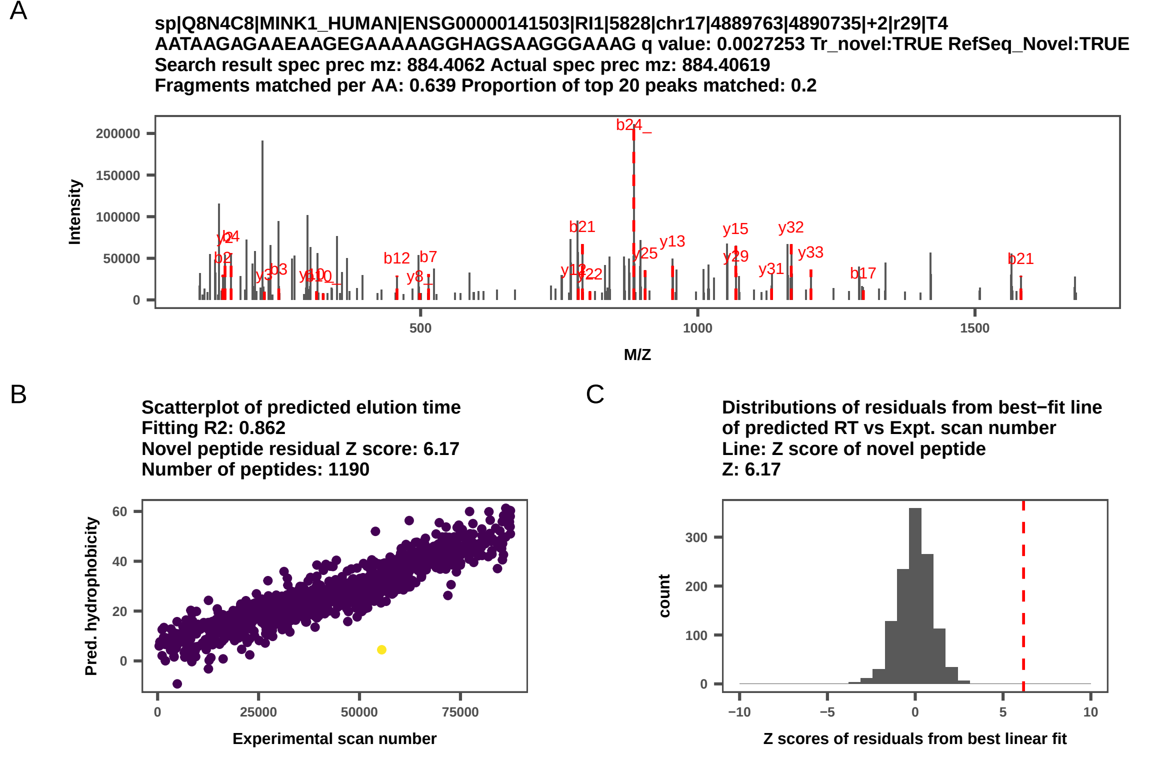
A
sp|Q8N4C8|MINK1_HUMAN|ENSG00000141503|RI1|5828|chr17|4889763|4890735|+2|r29|T4
AATAAGAGAAEAAGEGAAAAAGGHAGSAAGGGAAAG q value: 0.0027253 Tr_novel:TRUE RefSeq_Novel:TRUE
Search result spec prec mz: 884.4062 Actual spec prec mz: 884.40619
Fragments matched per AA: 0.639 Proportion of top 20 peaks matched: 0.2
Intensity
200000
150000
100000
50000
0
b24_
b21
y15
y32
y13
y25
y29
y33
y31
b17
b21
b12
b7
y8_
b2
b3
M/Z
500
1000
1500
B
Scatterplot of predicted elution time
Fitting R2: 0.862
Novel peptide residual Z score: 6.17
Number of peptides: 1190
Pred. hydrophobicity
60
40
20
0
0
25000
50000
75000
Experimental scan number
C
Distributions of residuals from best−fit line
of predicted RT vs Expt. scan number
Line: Z score of novel peptide
Z: 6.17
count
300
200
100
0
−10
−5
0
5
10
Z scores of residuals from best linear fit
